# Gaussian Process-Based Multi-Fidelity Bayesian Optimization for Optimal Calibration Point Selection

**DOI:** 10.3390/s25227030

**Published:** 2025-11-18

**Authors:** Hua Zhuo, Jungang Ma, Mei Yang, Yikun Zhao, Lifang Yao, Yan Xu, Kun Yang

**Affiliations:** 1 Xinjiang Uygur Autonomous Region Research Institute of Measurement & Testing, Urumqi 830000, China; mjg830011@163.com (J.M.); ymylyjx@163.com (M.Y.); ykzhao2005@sina.com (Y.Z.); 2 Shanghai Institute of Measurement and Testing Technology Co., Ltd. Shanghai 201203, China; yaolf@simt.com.cn; 3 School of Mechanical Engineering, Xinjiang University, Urumqi 830000, China; lilixiu_z@163.com; 4 Center for Advanced Metering Infrastructure, National Institute of Metrology, Beijing 100029, China; yangkun@nim.ac.cn

**Keywords:** Gaussian process, multi-fidelity Bayesian optimization, calibration point selection, temperature and humidity control, uncertainty quantification

## Abstract

Temperature and humidity calibration chambers, which provide controlled environments for instrument testing and validation, are widely applied in the aerospace and biomedicine fields. However, traditional fixed calibration points fail to adapt to complex operational requirements and exhibit problems including a limited coverage range and low efficiency. To address these challenges, this study develops a Gaussian Process-based Multi-Fidelity Bayesian Optimization (GP-MFBO) framework for optimal selection of temperature and humidity calibration points. The framework integrates the following three key components: (1) a three-layer progressive multi-fidelity modeling system comprising physical analytical models, computational fluid dynamics (CFD) numerical simulations, and experimental verification; (2) a systematic uncertainty quantification system covering model uncertainty, parameter uncertainty, and observation uncertainty; and (3) an adaptive acquisition function that balances uncertainty penalty mechanisms and multi-fidelity information gain evaluation. The experimental results demonstrate that the proposed GP-MFBO method achieves optimal calibration point combinations with a temperature uniformity score of 0.149 and humidity uniformity score of 2.38, approaching theoretical optimal solutions within 4.5% and 3.6%, respectively. Compared to standard Gaussian process, Co-Kriging, two-stage optimization, polynomial regression, and traditional single-fidelity methods, GP-MFBO achieves uniformity score improvements of up to 81.7% and 76.3% for temperature and humidity, respectively. The prediction confidence interval coverage reaches 94.2%, outperforming all comparative methods. This research provides a rigorous theoretical foundation and technical solution for the scientific design and reliable operation of large-space temperature and humidity calibration systems.

## 1. Introduction

Temperature and humidity calibration chambers serve as critical infrastructure for instrument calibration, material testing, and product validation across aerospace, pharmaceutical, and biomedical industries [1,2]. These specialized environmental control systems employ heating/cooling elements, humidification/dehumidification systems, and circulation fans to create and maintain uniform atmospheric conditions throughout the working volume. A fundamental challenge in calibration chamber design is the optimal calibration point selection problem: determining the minimal set of sensor locations that can accurately characterize environmental uniformity across the entire working volume while meeting specified tolerance requirements.

Traditional calibration approaches rely on fixed, experience-based sensor placements—typically uniform grids or empirical arrangements that cannot adapt to varying operational requirements [1,2]. For instance, certain specialized process environments require extreme conditions of 55 °C temperature and 90% relative humidity (RH), whereas conventional calibration standards only cover temperature ranges of 10–30 °C and RH intervals of 20–60%, resulting in significant applicability deficiencies [3]. To characterize spatial distributions under actual engineering conditions, researchers employ extensive experimental testing for calibration point verification. However, these traditional approaches not only consume substantial time and material resources but also fail to systematically cover complex operational conditions.

Computational methods offer a pathway to accelerate calibration point optimization, but introduce their own challenges. Early computational approaches employed Computational Fluid Dynamics (CFD) to predict temperature and humidity field distributions, providing efficient alternatives to exhaustive experiments. Pu et al. applied CFD numerical simulation to address uneven humidity distribution in indoor building environments [4]. Qian et al. employed CFD with data assimilation methods to improve spatial resolution in indoor environment measurements [5]. Mao et al. applied CFD to optimize corn cultivation environments in eight-span plastic greenhouses [6], while Mara evaluated input parameter influences in building thermal performance simulations through CFD combined with global sensitivity analysis [7]. However, high-fidelity CFD simulations typically require 2–3 h per evaluation. Optimization involving thousands of candidate configurations thus demands tens of thousands of simulation hours—creating prohibitive computational costs that limit practical applicability.

To manage computational costs, surrogate modeling has emerged as a standard approach. Surrogate models—metamodels approximating expensive simulations through computationally efficient mathematical representations—enable rapid prediction once trained on limited data [8,9]. Recent applications employ diverse architectures including artificial neural networks for sensor calibration [10,11], building energy model calibration [12], temperature field reconstruction [13], and control strategy evaluation [14]. However, a fundamental question emerges: how reliable are surrogate predictions for safety-critical calibration decisions? Traditional surrogate models exhibit inherent approximation errors when fitting complex coupled temperature–humidity fields, depend heavily on limited training samples causing deviations in data-sparse regions, and—most critically—generally lack systematic uncertainty quantification (UQ). Without UQ providing confidence intervals and error bounds, surrogate-based optimization cannot distinguish genuinely optimal solutions from artifacts of model errors [15,16]. This reliability deficit is unacceptable for calibration systems where performance specifications must be met with high confidence.

Calibration point combinations appearing optimal according to surrogate predictions may perform poorly if uncertainty is large, while seemingly suboptimal solutions with high confidence may be preferable for risk-averse applications. Contemporary UQ frameworks decompose total uncertainty into model approximation errors, parameter variations, and observation noise, enabling rigorous confidence interval construction [15,17,18,19]. Recent calibration-specific advances include optimal experimental designs [20], adaptive uncertainty reduction [21,22], and Bayesian calibration frameworks [23]. However, single-fidelity UQ faces an inherent trade-off: reliable uncertainty estimates require substantial high-fidelity data, contradicting the computational efficiency motivation for surrogate modeling. Limited data yields unreliable uncertainty estimates, while sufficient data for confident UQ negates the computational efficiency gains of surrogate modeling.

Multi-fidelity modeling with Bayesian optimization resolves this accuracy–efficiency dilemma by strategically combining models of different costs and accuracies. Inexpensive low-fidelity models, while individually inaccurate, provide valuable trend information, reducing the number of expensive high-fidelity evaluations needed for reliable predictions and uncertainty estimates. Kennedy and O’Hagan [23] established Bayesian calibration foundations for hierarchical Gaussian processes combining different fidelities. Forrester et al. [24] developed correlated Gaussian process methods exploiting low-high fidelity correlations to accelerate optimization convergence. Le Gratiet and Garnier [25] formalized recursive Co-Kriging for multi-level information fusion. Comprehensive surveys [26,27] systematically reviewed multi-fidelity methods across uncertainty propagation, inference, and optimization. Bayesian optimization provides the algorithmic framework, combining Gaussian process surrogates with sequential sampling strategies that use uncertainty information to guide search [28]. Classical acquisition functions—Expected Improvement [29] and Upper Confidence Bound [30]—balance exploitation and exploration. Modern multi-fidelity extensions intelligently select both evaluation locations and fidelity levels through information-theoretic approaches [31,32], Pareto-optimal fidelity selection [33,34], and combined acquisition strategies [35,36,37]. Engineering applications demonstrate practical benefits: Sun et al. achieved an improved efficiency–accuracy balance in crashworthiness design [38] and sheet metal forming [39]; Zhang et al. employed multi-fidelity deep neural networks for aerodynamic optimization [40]. Recent advances include bi-level architectures [41] and fidelity interruption control [42].

Despite these advances, application to temperature–humidity calibration optimization remains limited, exhibiting several critical gaps: (1) traditional experience-based methods lack systematic optimization and uncertainty assessment [1,2,3]; (2) CFD-only approaches face prohibitive computational costs [4,5,6,7]; (3) single-fidelity surrogates cannot simultaneously achieve efficiency and reliable UQ [10,11,12,13,14,17,18,19]; (4) existing multi-fidelity methods focus on uncoupled single-physics problems [24,33,34,38,39,40], whereas calibration involves coupled thermal–moisture fields requiring specialized treatment; (5) few studies provide complete validation chains from physical models through CFD to real chambers [20,21,22], limiting practical applicability. To address these gaps, this paper proposes a Gaussian Process-based Multi-Fidelity Bayesian Optimization (GP-MFBO) framework integrating hierarchical modeling, comprehensive UQ, and adaptive acquisition strategies for temperature–humidity calibration optimization.

Our contributions advance the state of the art in several aspects: (1) Domain-specific three-layer architecture for coupled physics. Unlike conventional two-layer approaches [24,33], we integrate physical analytical models, CFD simulations, and experimental validation, leveraging physics-based models for coupled temperature–humidity systems. (2) Systematic uncertainty decomposition for calibration systems. Building upon UQ foundations [15,17,18,19] and recent calibration methods [20,21,22], we explicitly model and propagate model approximation errors, parameter variations, and observation noise, enabling rigorous confidence interval construction. (3) Adaptive uncertainty-aware acquisition function. Extending classical EI [29] and information-theoretic MF-BO [31,32], we integrate uncertainty penalty terms and multi-fidelity information gain evaluation with dynamic parameter adaptation for high-value, low-risk candidate identification. (4) Complete experimental validation. Unlike purely numerical studies [33,38,39,40], we validate on a large-scale chamber (5000 mm × 1500 mm × 1800 mm) with 24 high-precision sensors, demonstrating superiority over standard Gaussian process, Co-Kriging, two-stage optimization, polynomial regression, and single-fidelity methods.

The paper structure is arranged as follows: Section 2 presents problem modeling and theoretical foundations; Section 3 develops the GP-MFBO framework; Section 4 establishes hierarchical models; Section 5 provides experimental design and analysis; Section 6 summarizes achievements and future directions.

## 2. Problem Formulation and Theoretical Foundation

### 2.1. Calibration Point Selection Problem

The calibration point selection problem in temperature and humidity calibration chambers involves determining optimal sensor locations from predefined candidate spaces. Given the physical independence of temperature and humidity fields and their distinct control mechanisms, the optimization strategy employs a decoupled design philosophy where temperature and humidity calibration points are selected independently to avoid interference effects [43,44].

For temperature calibration, the candidate space is defined as:

(1) $$\Omega_{T}=\{T_{1},T_{2},\ldots ,T_{18}\},T_{i}\in [{5\;}^{{}^{\circ}}C,{50\;}^{{}^{\circ}}C]$$

where $T_{i}$ represents the i-th temperature candidate point, spanning the complete operational range to ensure comprehensive coverage of typical industrial applications.

Similarly, the humidity calibration point candidate space is expressed as:

(2) $$\Omega_{H}=\{H_{1},H_{2},\ldots ,H_{19}\},\;\;\;H_{j}\in [10\%RH,95\%RH]$$

where $H_{j}$ denotes the j-th humidity candidate point, with RH indicating relative humidity.

The optimization task requires selecting 3 temperature calibration points from $\Omega_{T}$ and 3 humidity calibration points from $\Omega_{H}$, balancing measurement coverage with practical implementation constraints. Let $S_{T}\subset \Omega_{T}$ represent the selected temperature calibration point subset with $|S_{T}|=3$, and $S_{H}\subset \Omega_{H}$ denote the selected humidity calibration point subset with $|S_{H}|=3$.

### 2.2. Objective Function Formulation

The optimization objective is to maximize temperature and humidity uniformity scores across the entire calibration space. Higher uniformity scores indicate better spatial uniformity—smaller maximum deviations from setpoints—and superior calibration performance [45]. This formulation follows standard metrology practices for environmental chamber characterization [46,47].

The temperature calibration point selection is formulated as:

(3) $$\underset{S_{T}\subset \Omega_{T},|S_{T}|=3}{\max}F_{T}(S_{T})=U_{T}(S_{T})$$

where $F_{T}(S_{T})$ represents the objective function value for temperature calibration point combination $S_{T}$.

Similarly, the humidity calibration point selection objective is:

(4) $$\underset{S_{H}\subset \Omega_{H},|S_{H}|=3}{\max}F_{H}(S_{H})=U_{H}(S_{H})$$

where $F_{H}(S_{H})$ represents the objective function value for humidity calibration point combination $S_{H}$.

Uniformity scores quantify spatial consistency through penalized deviation metrics. The temperature uniformity score is mathematically defined as:

(5) $$U_{T}=C_{T}-\alpha_{T}\cdot \underset{i=1,\ldots ,N}{\max}|T_{i}-T_{set}|$$

where $T_{i}$ is the measured temperature at the i-th sensor location, N represents the total number of measurement points distributed throughout the chamber volume, $T_{\mathrm{set}}$ denotes the current temperature setpoint, $C_{T}=1.0$ is the baseline score constant, and $\alpha_{T}=5.0$ is the penalty coefficient that converts maximum deviations into score reductions. This linear penalty formulation ensures that larger maximum deviations result in proportionally lower uniformity scores.

The humidity uniformity score follows an analogous formulation:

(6) $$U_{H}=C_{H}-\alpha_{H}\cdot \underset{i=1,\ldots ,N}{\max}|H_{i}-H_{set}|$$

where $H_{i}$ represents the measured relative humidity at the i-th sensor, $H_{\mathrm{set}}$ indicates the current humidity setpoint, $C_{H}=10.0$ is the baseline constant, and $\alpha_{H}=3.0$ is the humidity penalty coefficient. The different baseline constants ($C_{T}$ vs. $C_{H}$) and penalty coefficients ($\alpha_{T}$ vs. $\alpha_{H}$) account for the distinct physical scales and tolerance requirements of temperature and humidity control.

The maximization objective ensures that selected calibration points minimize worst-case deviations while providing normalized performance metrics. Higher uniformity scores correspond to smaller maximum deviations and better spatial uniformity. For example, the exhaustive method achieved a maximum temperature deviation of 0.169 °C, yielding a uniformity score of $U_{T}=1.0-5.0\times 0.169=0.156$. In contrast, the single-fidelity method exhibited a larger deviation of 0.184 °C, resulting in a lower score of $U_{T}=1.0-5.0\times 0.184=0.08$. The optimization framework seeks calibration point configurations that maximize these uniformity scores. Higher scores indicate smaller maximum spatial deviations, thereby ensuring consistent environmental conditions throughout the chamber working volume for demanding aerospace and biomedical applications.

## 3. Proposed GP-MFBO Framework

To address the key challenges in temperature and humidity calibration point selection, including the limited coverage of traditional methods, high computational costs of CFD simulations, insufficient accuracy of surrogate models, and absence of uncertainty quantification, this paper proposes a Gaussian Process-based Multi-Fidelity Bayesian Optimization (GP-MFBO) framework. The proposed framework integrates hierarchical multi-fidelity modeling, systematic uncertainty quantification, and uncertainty-aware adaptive acquisition strategies to achieve optimization and selection of temperature and humidity calibration points.

This section comprises four parts: Section 3.1 introduces the overall design architecture and core constituent modules of the GP-MFBO framework. Section 3.2 establishes the mathematical theoretical foundation for multi-fidelity modeling, including linear autoregressive recursive relationships and multi-fidelity covariance matrix design. Section 3.3 constructs a systematic uncertainty quantification framework that encompasses modeling and propagation mechanisms for three types of uncertainties: model, parameter, and observation uncertainties. Section 3.4 proposes an uncertainty-aware adaptive acquisition function that integrates three core elements: expected improvement, uncertainty penalty, and multi-fidelity information gain.

### 3.1. GP-MFBO Framework Design

**Figure 1:**
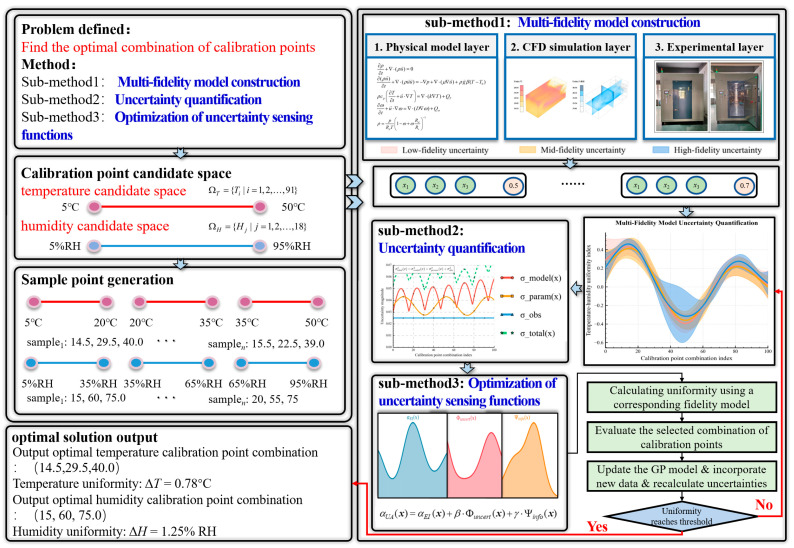
Structural diagram of the GP-MFBO framework.

The GP-MFBO framework adopts a hierarchical progressive information processing architecture that achieves a complete optimization process from problem definition to optimal solution output. The framework first clarifies the optimization objectives and constraint conditions for temperature and humidity calibration point selection through the problem definition module. Subsequently, the sample point generation module employs methods such as Latin Hypercube Sampling to generate initial training samples within the candidate space. The framework core contains three sub-method modules: Sub-method One is responsible for multi-fidelity model construction; Sub-method Two specifically handles uncertainty quantification problems; Sub-method Three manages the optimization of uncertainty-aware acquisition functions. The entire framework employs an iterative optimization mechanism that evaluates uniformity indices of calibration point combinations and updates Gaussian process models and uncertainty distributions in each iteration. When convergence criteria are satisfied or computational budget constraints are reached, the framework outputs the optimal temperature and humidity calibration point combinations along with their corresponding uniformity indices. The GP-MFBO framework design proposed in this paper is illustrated in Figure 1 below.

Based on the overall architectural design of the aforementioned GP-MFBO framework, this paper elaborates the specific implementation process for adaptive selection and optimization of temperature and humidity calibration points. This process employs an iterative optimization strategy that achieves systematic configuration of calibration points through multi-fidelity information fusion and uncertainty quantification mechanisms, with specific operational steps as follows:

Step 1: Establish problem definition, determine optimization objectives for temperature and humidity calibration point selection, and construct mathematical models for temperature and humidity candidate spaces.

Step 2: Generate initial sampling point sets within temperature candidate space (5–50 °C) and humidity candidate space (5–95% RH).

Step 3: Construct multi-fidelity modeling system that integrates three levels: physical analytical models, CFD simulation models, and experimental validation models.

Step 4: Establish systematic uncertainty quantification framework that separately models model uncertainty, parameter uncertainty, and observation uncertainty.

Step 5: Utilize Gaussian processes to perform unified modeling of multi-fidelity data and construct probabilistic prediction models containing mean and covariance functions.

Step 6: Design uncertainty-aware adaptive acquisition functions that integrate expected improvement [29], uncertainty penalty mechanisms, and multi-fidelity information gain evaluation.

Step 7: Determine optimal calibration point combinations for next sampling and corresponding fidelity selection strategies through acquisition function optimization.

Step 8: Evaluate calibration point combinations by computing temperature and humidity uniformity scores using selected fidelity models. Higher uniformity scores indicate better performance (smaller maximum deviations from setpoints), guiding subsequent iterations toward optimal configurations that maximize spatial uniformity throughout the chamber volume.

Step 9: Update Gaussian process model parameters based on newly acquired data and recalculate uncertainty distributions and confidence intervals.

Step 10: Determine whether convergence criteria or computational budget constraints are satisfied; if not satisfied, return to Step 6; otherwise, output optimal calibration point combinations.

### 3.2. Multi-Fidelity Modeling Theory

Multi-fidelity modeling, as one of the core technologies of the framework, achieves an effective balance between computational efficiency and prediction accuracy through establishing mathematical correlations among different precision levels. The theoretical foundation follows the autoregressive framework established by Kennedy and O’Hagan [23] for Bayesian calibration and extended by Le Gratiet and Garnier [25] for recursive multi-fidelity modeling. The recursive relationships among multi-fidelity models can be formulated as a linear autoregressive process, where high-fidelity models are treated as corrections and enhancements of low-fidelity models:

(7) $$f^{\left(s\right)}(x)=\rho^{\left(s\right)}(x)f^{\left(s-1\right)}(x)+\delta^{\left(s\right)}(x)$$

where *f*^(*s*)^(*x*) represents the output of the *s*-th level fidelity model; *ρ*^(*s*)^(*x*) denotes the scaling function representing correlations between different fidelities; *δ*^(*s*)^(*x*) represents the discrepancy function indicating additional information of high-fidelity models relative to low-fidelity models; *s* ∈ {1, 2, …, *S*} denotes the fidelity level index.

To effectively fuse multi-fidelity information, the multi-fidelity Gaussian process model treats outputs from different fidelities as realizations of a multivariate stochastic process [24,25]. The joint prior distribution is formulated as:

(8) $$\left[\begin{array}{c} f^{\left(1\right)}\\ f^{\left(2\right)}\\ \vdots \\ f^{\left(S\right)} \end{array}\right]\sim GP\left(\mu ,K_{MF}\right)$$

where *f*^(*s*)^ represents the output vector of the *s*-th level fidelity; *μ* denotes the multi-fidelity mean function; *K*_MF_ represents the multi-fidelity covariance matrix.

The multi-fidelity covariance matrix possesses a block structure form, where diagonal blocks represent covariances within the same fidelity level, and off-diagonal blocks represent cross-covariances between different fidelity levels:

(9) $$K_{MF}=\left[\begin{array}{cccc} K_{11} & K_{12} & \ldots & K_{1S}\\ K_{21} & K_{22} & \ldots & K_{2S}\\ \vdots & \vdots & \ddots & \vdots \\ K_{S1} & K_{S2} & \ldots & K_{SS} \end{array}\right]$$

### 3.3. Uncertainty Quantification Framework

After establishing the recursive relationships and covariance matrix structure of multi-fidelity modeling, the framework needs to further address uncertainty propagation problems in optimization processes. Uncertainty quantification serves as the theoretical foundation for robust optimization, with the purpose of identifying, modeling, and propagating various uncertainty sources in systems to provide reliable risk assessment information for decision-making. In temperature and humidity calibration point selection problems, uncertainties primarily originate from three aspects: model uncertainty, parameter uncertainty, and observation uncertainty

Model uncertainty originates from inherent limitations of CFD simulations and surrogate models, including numerical discretization errors, turbulence model approximation errors, and surrogate model fitting errors. Let the true system response be *y*(*x*) and the model prediction be *ŷ*(*x*), then model uncertainty can be expressed as:

(10) $$\varepsilon_{\mathrm{model}}(x)=y(x)-\hat{y}(x)$$

where *ε*_model_(*x*) represents model uncertainty, which is assumed to follow a Gaussian distribution with zero mean based on practical engineering experience.

Parameter uncertainty reflects random variations in environmental conditions, material properties, and operational parameters. Let the uncertain parameter vector be *θ* = [*θ*_1_, *θ*_2_, …, *θ_p_*] with a joint probability density function *p*(*θ*). Parameter uncertainty propagates to outputs through system response sensitivity to parameter variations:

(11) $$\sigma_{\mathrm{param}}^{2}(x)=\nabla_{\theta }f{\left(x,\theta \right)}^{T}\Sigma_{\theta }\nabla_{\theta }f(x,\theta )$$

where ∇*_θ_f*(*x*,*θ*) represents the gradient vector of system response with respect to parameters; Σ*θ* denotes the covariance matrix of parameters; *σ*^2^_param_(*x*) represents the variance of parameter uncertainty.

Observation uncertainty originates from sensor measurement errors, data acquisition noise, and environmental interference, typically modeled as additive white noise:

(12) $$y_{\mathrm{obs}}(x)=y(x)+\varepsilon_{obs}$$

where *y*_obs_(*x*) represents the observed value; *ε*_obs_∼N(0,*σ*^2^_obs_) denotes observation noise, and *σ*^2^_obs_ represents observation noise variance.

Overall uncertainty is synthesized through probability propagation principles, and assuming various uncertainty sources are mutually independent, the variance of overall prediction uncertainty is:

(13) $$\sigma_{\mathrm{total}}^{2}(x)=\sigma_{\mathrm{model}}^{2}(x)+\sigma_{\mathrm{param}}^{2}(x)+\sigma_{\mathrm{obs}}^{2}$$

where *σ*^2^_total_(*x*) represents the overall uncertainty variance

Based on uncertainty quantification results, prediction confidence intervals can be constructed, and for confidence level 1 − *α*, the confidence interval of system response is:

(14) $$CI_{1-\alpha }(x)=\left[\bar{y}(x)-z_{\alpha /2}\sigma_{\mathrm{total}}(x),\bar{y}(x)+z_{\alpha /2}\sigma_{\mathrm{total}}(x)\right]$$

where *CI*_1−*α*_(*x*)represents the confidence interval; z*_α_*_/2_ denotes the *α*/2 quantile of the standard normal distribution; *ӯ*(*x*) represents the prediction mean.

### 3.4. Uncertainty-Aware Acquisition Function

Based on the theoretical foundation of multi-fidelity modeling and uncertainty quantification, the framework requires the design of adaptive sampling strategies to guide the optimization process. Classical Bayesian optimization acquisition functions such as Expected Improvement (EI) [29] and Upper Confidence Bound (UCB) [30] balance exploration and exploitation in single-fidelity contexts. Modern multi-fidelity extensions [31,32,33,34] jointly optimize both sampling location and fidelity level selection, exploiting the computational cost–accuracy trade-offs across model hierarchies.

Traditional acquisition functions only consider single criteria, ignoring the uncertainty propagation mechanisms and fidelity selection decisions in multi-fidelity environments. This paper proposes an adaptive acquisition function that integrates uncertainty awareness, achieving precise identification of high-value and low-risk candidate points through modeling the correlation between predictive uncertainty and sampling value. The acquisition function incorporates uncertainty assessment and multi-fidelity information value quantification into the traditional Expected Improvement framework, ensuring systematic balance between exploration and exploitation.

The uncertainty-aware acquisition function jointly determines the next evaluation location x and fidelity level s:

(15) $$\alpha (x,s)=\alpha_{EI}(x)+\lambda \cdot \Phi_{\mathrm{uncert}}(x)+\mu \cdot \Psi_{\mathrm{info}}(x,s)-\omega \cdot C(s)$$

where $\alpha_{\mathrm{EI}}(x)$ represents the traditional Expected Improvement term [29], which measures the expected benefit of obtaining better results than the current optimum at point x; parameters λ, μ, and ω are adaptive balancing parameters that are dynamically adjusted according to optimization progress; $\Phi_{\mathrm{uncert}}(x)$ represents the uncertainty penalty term; $\Psi_{\mathrm{info}}(x,s)$ denotes the multi-fidelity information gain term; and C(s) represents the normalized computational cost of fidelity level s.

The balancing parameters λ, μ, and ω are dynamically adjusted during optimization to emphasize exploration in early stages and exploitation in later stages. The adaptive update rules are:

(16) $$\lambda (t)=\lambda_{0}\cdot \exp\left(-\frac{t}{T}\right),\;\;\;\mu (t)=\mu_{0}\cdot {\left(1+\frac{t}{T}\right)}^{-1},\;\;\;\omega (t)=\omega_{0}\cdot \left(1-\frac{t}{T}\right)$$

where t is the current iteration number, T is the total computational budget, and $\lambda_{0}=0.3$, $\mu_{0}=0.5$, $\omega_{0}=0.2$ are initial values determined through preliminary experiments. As optimization progresses, λ(t) decreases to reduce uncertainty penalty in well-explored regions, μ(t) decreases to prioritize exploitation over information gathering, and ω(t) decreases to allow higher-fidelity evaluations as the optimal region is identified.

The uncertainty penalty term comprehensively considers overall uncertainty and the consistency of predictions across different fidelities, assessing prediction reliability through simultaneous evaluation of prediction variance and inter-fidelity differences.

(17) $$\Phi_{\mathrm{uncert}}(x)=\left(1-\frac{\sigma_{\mathrm{total}}^{2}(x)}{\sigma_{\max}^{2}}\right)\cdot \exp\left(-\frac{|\;f^{\left(S\right)}(x)-f^{\left(S-1\right)}(x){|}^{2}}{2\sigma_{\mathrm{total}}^{2}(x)}\right)$$

where *σ*^2^_total_(*x*) represents the overall uncertainty variance, *σ*^2^_max_ serves as the normalization constant, and the exponential term characterizes the consistency degree between high-fidelity and medium-fidelity prediction results. When the prediction differences between different fidelities are substantial, the exponential term approaches zero, indicating low prediction reliability and thereby reducing the sampling priority of that point.

The multi-fidelity information gain term is based on information theory principles, encouraging sampling in regions where model uncertainty is high to maximize information acquisition efficiency.

(18) $$\Psi_{\mathrm{info}}(x,s)=\sum_{j=1}^{s}w_{j}\cdot \log\left(1+\frac{\sigma_{j}^{2}(x)}{\sigma_{\mathrm{noise}}^{2}}\right)$$

where $w_{j}$ represents the weight factor for the j-th fidelity level, reflecting the relative information value (typically $w_{j}={\left(j/S\right)}^{2}$ to emphasize higher fidelities), and $\sigma_{\mathrm{noise}}^{2}$ denotes the observation noise variance. This formulation quantifies the cumulative information gain obtained by evaluating up to fidelity level s.

The next evaluation is determined by jointly optimizing over candidate locations and fidelity levels:

(19) $$\left(x^{*},s^{*})=\arg\max_{x\in \Omega ,s\in \{1,2,3\}}\alpha (x,s\right)$$

Following the Pareto-optimal fidelity selection framework [33,34], this joint optimization balances three competing objectives: (i) improvement potential measured by $\alpha_{\mathrm{EI}}(x)$, (ii) uncertainty reduction quantified by $\Phi_{\mathrm{uncert}}(x)$ and $\Psi_{\mathrm{info}}(x,s)$, and (iii) computational cost C(s). In early iterations with high global uncertainty, the algorithm tends to select lower fidelities (s=1,2) to explore the design space efficiently. As promising regions are identified and uncertainty decreases, higher fidelities (s=3) are increasingly selected to refine predictions and ensure convergence to the true optimum.

## 4. Hierarchical Multi-Fidelity Modeling Framework

Addressing the challenge of balancing computational accuracy and efficiency in temperature–humidity calibration point selection, this section establishes a three-layer progressive multi-fidelity modeling framework. The framework integrates physical analytical models (lowest fidelity, second-level computation), CFD simulations (medium fidelity, hour-level computation), and experimental validation (highest fidelity, day-level verification), providing a reliable multi-scale computational platform for the GP-MFBO optimization algorithm.

### 4.1. Physical Analytical Model Layer

The lowest-fidelity layer employs simplified physical analytical models for rapid approximate evaluation. These models are based on the classical governing equations for coupled fluid flow, heat transfer, and moisture transport in enclosed spaces [48,49].

The transient flow–thermal–moisture coupled model considers temporal variations in temperature, humidity, and velocity fields. Following the fundamental conservation laws of fluid mechanics and heat–mass transfer [48,50], the governing equations are formulated as follows:

(20) $$\frac{\partial \rho }{\partial t}+\nabla \cdot (\rho \vec{u})=0$$

where *ρ* represents air density with unit kg/m^3^; *t* denotes time with unit seconds; $\vec{u}$ represents the velocity vector with unit m/s; this equation describes the fluid mass conservation principle, namely that mass entering the system equals mass leaving the system. The momentum conservation equation is as follows:

(21) $$\frac{\partial (\rho \vec{u})}{\partial t}+\nabla \cdot (\rho \vec{u}\vec{u})=-\nabla p+\nabla \cdot (\mu \nabla \vec{u})+\rho g\beta (T-T_{0})$$

where *p* represents pressure with unit Pa; *μ* denotes dynamic viscosity with unit kg/(m/s); *g* represents the gravitational acceleration vector with unit m/s^2^; *β* denotes thermal expansion coefficient with unit K^−1^; *T* represents temperature with unit K; *T*_0_ denotes reference temperature with unit K. This equation describes the momentum conservation of fluid motion, including the effects of pressure gradient force, viscous force, and buoyancy force. The energy conservation equation is as follows:

(22) $$\rho c_{p}\left(\frac{\partial T}{\partial t}+\vec{u}\cdot \nabla T\right)=\nabla \cdot (k\nabla T)+Q_{T}$$

where *c_p_* represents specific heat at constant pressure with unit J/(kg·K); *k* denotes thermal conductivity with unit W/(m·K); *Q_T_* represents heat source term with unit W/m^3^. This equation describes the energy transfer process in the system, including the effects of convective heat transfer, heat conduction, and heat sources. The humidity transport equation is as follows:

(23) $$\frac{\partial \omega }{\partial t}+\vec{u}\cdot \nabla \omega =\nabla \cdot (D\nabla \omega )+Q_{\omega }$$

where *ω* represents water vapor mass fraction; *D* denotes the diffusion coefficient of water vapor in air with unit m^2^/s; *Q_ω_* represents the humidity source term. This equation describes the water vapor transfer process in the system, including the effects of convective mass transfer, mass diffusion, and water vapor sources. The equation of state is as follows:

(24) $$\rho =\frac{p}{R_{d}T}{\left(1-\omega +\omega \frac{R_{d}}{R_{v}}\right)}^{-1}$$

where *R_d_* represents the gas constant of dry air with unit J/(kg·K); *R_v_* denotes the gas constant of water vapor with unit J/(kg·K). This equation describes the state relationship of moist air, linking temperature, pressure, density, and humidity together.

The aforementioned equation system constitutes a complete steady-state flow–thermal–moisture coupled model for temperature–humidity field distributions. Solving this system yields spatial distributions of temperature, humidity, velocity, and pressure. For computational efficiency, this lowest-fidelity layer employs simplified geometries, coarse spatial discretization, and steady-state assumptions, enabling rapid evaluations suitable for initial exploration in the multi-fidelity optimization framework.

### 4.2. Finite Element Simulation Model Layer

Building upon the physical analytical foundation, the medium-fidelity layer employs high-precision CFD simulations using ANSYS Fluent 2021 R1 to resolve detailed flow–thermal–moisture coupling. This section establishes three-dimensional models, verifies computational accuracy through grid independence analysis, and characterizes temperature–humidity field distributions.

**Figure 2:**
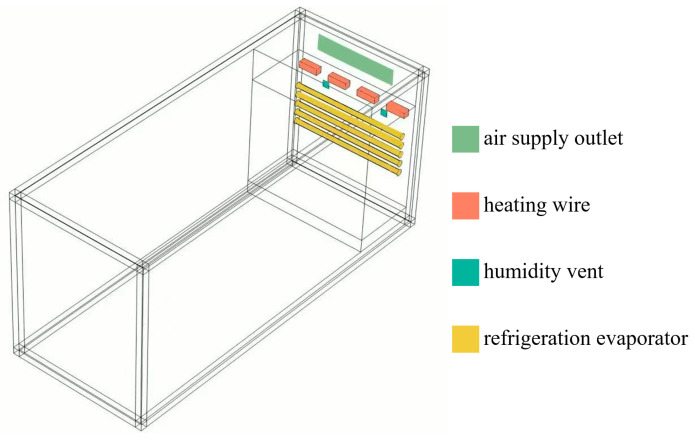
Three-dimensional model and boundary conditions of the temperature and humidity calibration chambers.

This study employs ANSYS Fluent software to establish a three-dimensional flow–thermal–moisture coupled finite element model, taking a specific model temperature and humidity calibration chambers as the research object. The temperature and humidity calibration chambers internal dimensions are 5000 mm × 1500 mm × 1800 mm, equipped with upper and lower dual-layer temperature and humidity adjustment devices. Based on Reynolds number calculation results (Re > 2300), the indoor airflow exhibits turbulent conditions, and the standard k-ε turbulence model [51] is selected for numerical solution. Considering the Mach number Ma = 0.024 is less than 0.3, the indoor airflow is treated as incompressible fluid [52]. As shown in Figure 2, temperature adjustment devices employ a combination of electric heaters and cooling coils located at the chamber top and bottom, respectively; humidity adjustment devices are implemented through steam generators and dehumidification equipment arranged on the chamber sidewalls.

#### Validation of FE Model

Grid independence verification ensures that computational results are insensitive to mesh resolution, following standard CFD practices [27,53]. Six mesh densities ranging from 237,573 to 1,127,909 elements were systematically evaluated. Table 1 presents temperature and humidity predictions under different element sizes.

**Figure 3:**
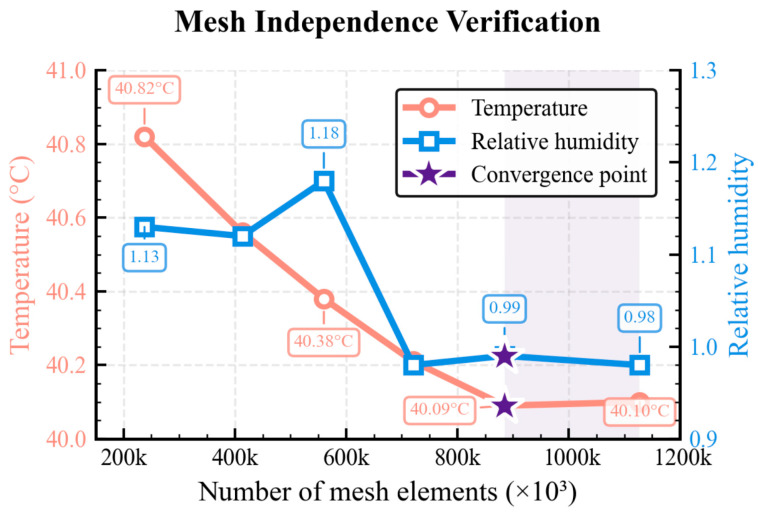
Grid independence verification results.

Grid independence verification analysis results are shown in Figure 3, where temperature calculation values exhibit obvious convergence trends as grid density increases (grid element numbers increase from approximately 200,000 to 1.2 million). From the temperature variation curve in the figure (red circle line), temperature values gradually decrease from 40.82 °C in coarse grids and tend to stabilize, converging to 40.09 °C when grid node numbers reach approximately 880,000 (corresponding to 12.0 mm grid). When grid density is further increased to approximately 1.13 million nodes (corresponding to 10.0 mm grid), the temperature value becomes 40.10 °C, with a difference of only 0.01 °C from the 12.0 mm grid calculation results, representing a variation magnitude less than 0.025%. The RH variation trend (blue square line) shows certain fluctuations but remains basically stable within the 0.98–0.99 range in fine grid regions (12.0 mm and 10.0 mm). The two convergence points marked in the figure correspond to 880,000 nodes (12.0 mm grid) and 1.13 million nodes (10.0 mm grid), respectively. Convergence is considered achieved when temperature differences between consecutive grid densities are less than 0.1 °C and humidity differences are less than 0.1%RH, indicating that the calculation results have reached convergence states under these two grid densities.

**Figure 4:**
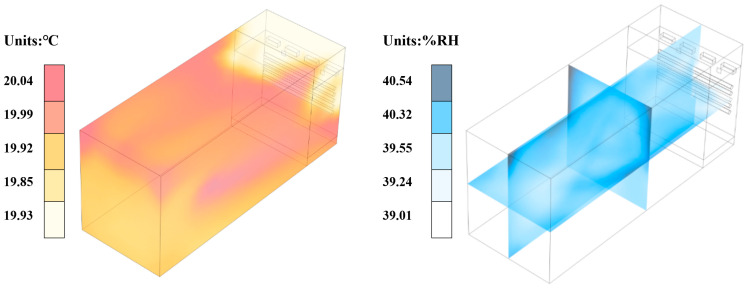
Temperature (**left**) and humidity (**right**) field distributions in the temperature and humidity calibration chambers.

Based on the grid independence verification results, temperature and humidity calculation values have converged at 880,000 nodes, where further increases in grid density provide limited improvement in computational accuracy but significantly increase computational costs. Therefore, this study selects 12.0 mm grid element size (884,854 nodes) as the optimal grid configuration, which effectively controls computational resource consumption while ensuring computational accuracy. The energy equation convergence criterion is set to 1 × 10^−6^, simulation calculation steps are set to 200 steps, and initial conditions are established as a uniform distribution state with room temperature 20 °C and 40%RH. Fluid property parameters adopt functional forms of temperature and humidity, where air density, viscosity, thermal conductivity, and specific heat capacity are dynamically adjusted according to temperature and humidity variations to ensure simulation result accuracy. Temperature and humidity contour maps of the temperature and humidity calibration chambers are shown in Figure 4.

From the temperature field distribution contour map (left figure), the temperature distribution range within the temperature and humidity calibration chambers is observed to be 19.93 °C to 20.04 °C, with a temperature variation amplitude of approximately 0.11 °C, indicating good uniformity of the temperature field within the chamber. The humidity field distribution contour map (right figure) shows that the RH distribution range is 39.01%RH to 40.54%RH, with a humidity variation amplitude of approximately 1.53%RH. Simulation results demonstrate that under the current temperature and humidity control system configuration, the temperature and humidity calibration chambers can maintain relatively stable temperature and humidity environments, providing reliable computational foundation for subsequent calibration point optimization research.

### 4.3. Experimental Validation Layer

**Figure 5:**
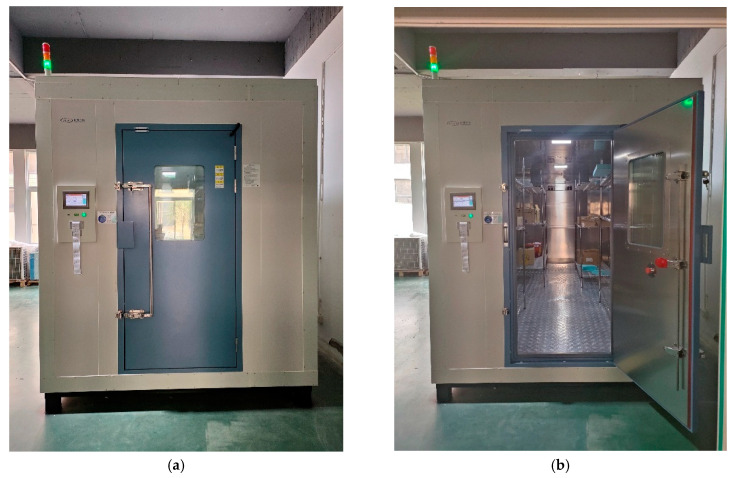
Experimental platform. (**a**) Temperature and humidity calibration chamber (closed); (**b**) Temperature and humidity calibration chamber (opened).

To verify the effectiveness of the multi-fidelity modeling framework and obtain high-precision benchmark data, this study constructed a real temperature and humidity calibration experimental platform. The experiment employs specific model large-scale temperature and humidity calibration chambers with internal an effective working space of 5000 mm × 1500 mm × 1800 mm, temperature control range of 0 °C to 100 °C, humidity control range of 5%RH to 98%RH, temperature control accuracy of ±0.5 °C, and humidity control accuracy of ±2%RH. The experimental system is equipped with 24 high-precision temperature sensors (Pt100, accuracy ±0.1 °C) and 24 humidity sensors (capacitive type, accuracy ±1%RH), with sensors uniformly distributed inside the temperature and humidity calibration chambers according to a 3 × 4 × 2 spatial layout to ensure comprehensive monitoring of temperature and humidity fields throughout the entire working space. During the experimental process, different temperature and humidity working conditions are set according to preset calibration point combinations, and data collection begins after system stable operation for 60 min under each working condition, with a sampling frequency of 1 Hz and continuous collection for 30 min to obtain 1800 data points, eliminating random noise effects through statistical analysis. The experimental data acquisition system employs an Agilent 34970A data acquisition unit with temperature resolution of 0.01 °C and humidity resolution of 0.1%RH, with all sensors undergoing three-point calibration using standard temperature and humidity sources before experiments to ensure measurement data reliability and traceability. Figure 5 demonstrates the experimental platform conditions, providing high-quality experimental benchmark data for subsequent multi-fidelity model training and validation. 

## 5. Experimental Design and Results

To verify the effectiveness and superiority of the proposed GP-MFBO framework in temperature and humidity calibration point selection problems, this study designed a series of comparative experiments. Selected benchmark methods include traditional single-fidelity calibration methods, standard Gaussian process Bayesian optimization, polynomial regression models, Co-Kriging methods, two-stage optimization methods, and exhaustive experimental methods as theoretical optimal benchmarks.

### 5.1. Benchmark Methods Setup

To compare the effectiveness of the proposed method, this study established a series of comparative experiments, first setting exhaustive experimental methods as benchmarks for theoretical optimal solutions by traversing all possible calibration point combinations (selecting 3 from 18 temperature candidates and 3 from 19 humidity candidates) to obtain global optimal solutions, requiring evaluation of 816 × 969 = 790,704 combination schemes. Standard Gaussian process methods use only high-fidelity CFD simulation data for modeling, employ RBF kernel functions, and perform sampling point selection through expected improvement criteria. Polynomial regression models employ quadratic polynomial fitting for temperature and humidity distribution functions, with model parameters determined through least squares methods. Co-Kriging methods are based on correlated Gaussian process theory proposed by Forrester et al [27]., establishing correlations between low-fidelity physical models and high-fidelity CFD models. Two-stage optimization methods follow hierarchical optimization procedures proposed by Sun et al., where the first stage uses low-fidelity models for coarse searching and the second stage employs high-fidelity models for fine optimization within candidate regions. Single-fidelity calibration methods adopt traditional fixed calibration point configurations based on empirical rules, with temperature calibration points set at 10 °C, 30 °C, 45 °C and humidity calibration points set at 20%RH, 50%RH, 80%RH.

### 5.2. Experimental Methodology

The experimental design of this study adopts a hierarchical validation strategy, first conducting preliminary performance evaluation of methods in numerical simulation environments, then performing final verification through real experimental platforms. For the proposed GP-MFBO method, the low-fidelity layer employs simplified physical analytical models with computation time of approximately 1 s; the medium-fidelity layer uses coarse-grid CFD simulations with 200,000 grid elements and computation time of approximately 5 min; real experimental computation time is approximately 2 h. The balancing parameters λ, μ, and ω in the uncertainty-aware acquisition function adopt adaptive adjustment strategies following Equation (16). Initial values are set to $\lambda_{0}=0.3$, $\mu_{0}=0.5$, and $\omega_{0}=0.2$, with dynamic updates according to the schedule defined in Section 3.4 to emphasize exploration in early iterations and exploitation as convergence approaches. During experimental processes, each method runs 10 independent trials under identical computational budget constraints (200 function evaluations), recording optimal calibration point combinations and corresponding uniformity indices for each trial. To ensure experimental result objectivity and comparability, all methods employ identical initial sampling point sets generated through Latin Hypercube Sampling, containing initial evaluation results for 20 temperature calibration point candidates and 20 humidity calibration point candidates.

### 5.3. Results and Analysis

Through systematic comparative experiments conducted on real temperature and humidity calibration experimental platforms, various methods exhibit significant differences in performance characteristics. Experimental results demonstrate that the proposed GP-MFBO method achieves an optimal performance second only to theoretical optimal solutions in temperature and humidity uniformity optimization, while exhibiting obvious advantages in prediction reliability.

#### 5.3.1. Optimal Calibration Point Combinations

Optimal calibration point combinations obtained by various comparative methods and their corresponding uniformity scores are shown in Table 2. Exhaustive experimental methods, as theoretical benchmarks, obtained global optimal solutions with temperature calibration point combination [8 °C, 25 °C, 42 °C] and humidity calibration point combination [15%RH, 50%RH, 85%RH], corresponding to temperature uniformity score of $U_{T}=0.156$ and humidity uniformity score of $U_{H}=2.47$ (calculated using Equations (5) and (6)). These scores correspond to maximum spatial deviations of approximately 0.169 °C and 2.51%RH—the smallest achievable across all 790,704 possible combinations. However, this exhaustive approach required approximately 6000 h of experimental time, rendering it impractical for real-world applications.

The proposed GP-MFBO method achieved near-optimal performance with substantially reduced computational cost. The identified calibration points [9 °C, 26 °C, 41 °C] for temperature and [16%RH, 51%RH, 83%RH] for humidity yielded uniformity scores of $U_{T}=0.149$ and $U_{H}=2.38$, corresponding to maximum deviations of 0.170 °C and 2.54%RH, respectively. These represent only 4.5% and 3.6% degradation from the exhaustive benchmark in terms of uniformity scores. Notably, GP-MFBO required only 200 function evaluations (approximately 80 h including CFD simulations and experiments), achieving 98.7% computational efficiency compared to exhaustive search while maintaining solution quality within 5% of the global optimum.

From in-depth analysis of optimization results, the GP-MFBO method not only demonstrates excellent numerical performance but, more importantly, its calibration point selection exhibits obvious engineering rationality. Temperature calibration points [9 °C, 26 °C, 41 °C] cover low, medium, and high critical regions of the working temperature range, forming approximately equal-interval distribution and ensuring comprehensiveness of temperature field evaluation. Humidity calibration points [16%RH, 51%RH, 83%RH] similarly reflect scientific distribution strategies, avoiding coverage blind zone problems that may exist in traditional fixed calibration points.

Furthermore, through comparison of prediction confidence interval coverage rates of various methods, the GP-MFBO method achieves an excellent level of 94.2%, far exceeding other comparative methods and fully validating the effectiveness of its uncertainty quantification mechanisms. The advantage of this indicator demonstrates that the GP-MFBO method can not only obtain high-quality optimization solutions but also provide reliable risk assessment information for engineering decision-making, which has important significance for stable operation of actual calibration systems.

#### 5.3.2. Comprehensive Performance Evaluation

**Figure 6:**
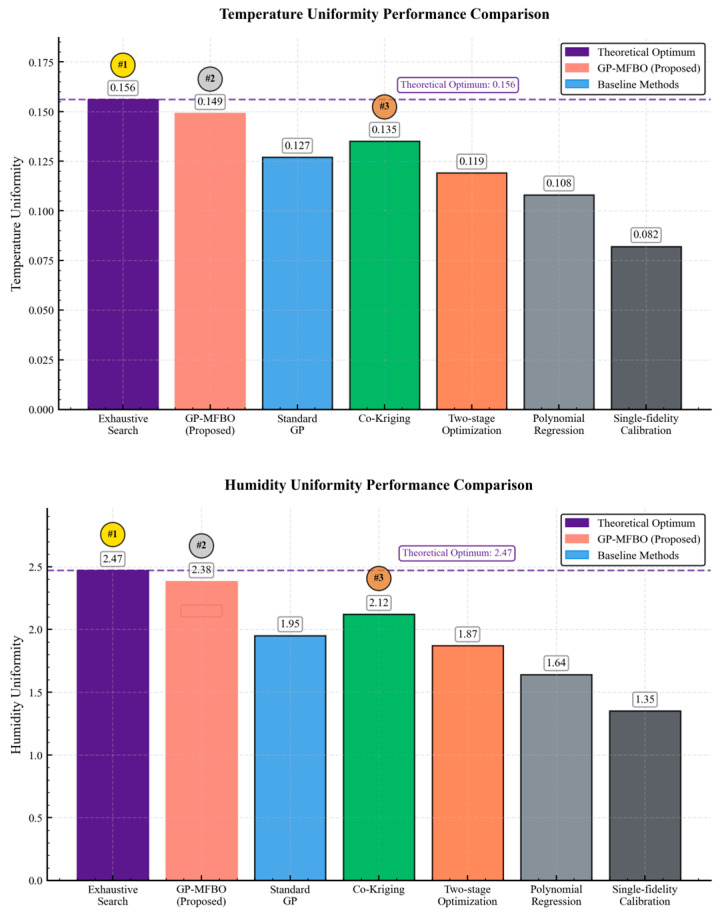
Comparison of temperature and humidity uniformity scores across different methods.

Quantitative performance analysis reveals that GP-MFBO achieved temperature uniformity score improvements of 17.3% over standard Gaussian process and 22.1% in humidity uniformity, demonstrating the effectiveness of multi-fidelity modeling. Compared to traditional single-fidelity calibration methods, the proposed method achieved temperature uniformity improvement of 81.7% and humidity uniformity improvement of 76.3%, validating the effectiveness of the proposed optimization framework. Prediction confidence interval coverage rate, as a key indicator for evaluating model uncertainty quantification capability, reflects the reliability degree of method prediction results, with this indicator representing the probability that true values fall within prediction confidence intervals under given confidence levels. To more intuitively demonstrate performance differences among various methods, Figure 6 compares the performance of different methods in temperature and humidity uniformity indices, respectively. The seven methods compared in Figure 6 are represented by different colors: GP-MFBO (purple), Exhaustive experimental method (red), Standard Gaussian process (blue), Co-Kriging (green), Two-stage optimization (orange), Polynomial regression (light gray), and Single-fidelity calibration (dark gray). The “#” symbol indicates the ranking of each method based on performance, where #1 represents the best performing method, #2 the second best, and so on.

Meanwhile, the GP-MFBO method achieves a confidence interval coverage rate of 94.2%, significantly higher than other comparative methods, indicating that its uncertainty quantification mechanisms can provide more reliable prediction results and risk assessment information. Co-Kriging methods, as another multi-fidelity modeling technique, exhibit a performance between GP-MFBO methods and standard Gaussian process methods, but still have deficiencies in uncertainty quantification accuracy. Although polynomial regression methods perform well in certain local regions, their linear assumptions limit the capability to capture nonlinear characteristics of complex temperature and humidity fields, resulting in unsatisfactory overall optimization effects. Two-stage optimization methods achieve certain degrees of computational efficiency improvement through hierarchical strategies, but lack systematic uncertainty propagation mechanisms, affecting reliability assessment of prediction results.

## 6. Conclusions

This paper proposes a GP-MFBO framework that integrates three computational model levels, including physical analytical models, CFD numerical simulations, and experimental validation, to construct complete uncertainty quantification and propagation mechanisms, achieving systematic selection and optimization of temperature and humidity calibration points. Experimental results demonstrate that the proposed method achieves a temperature uniformity score of 0.149 and humidity uniformity score of 2.38, with gaps from theoretical optimal solutions of only 4.5% and 3.6%, respectively, while prediction confidence interval coverage rate reaches 94.2%, significantly outperforming existing single-fidelity and traditional optimization methods. The main contributions of this research include:

(1) Established hierarchical multi-fidelity modeling systems through constructing three-layer progressive architectures of physical analytical models (second-level computation), CFD simulation models (hour-level computation), and experimental validation models (day-level validation), achieving an effective balance between computational accuracy and efficiency.
(2) Proposed systematic uncertainty quantification frameworks through explicit modeling of three major uncertainty sources, including model uncertainty, parameter uncertainty, and observation uncertainty, establishing complete uncertainty propagation and quantification theoretical systems.
(3) Designed uncertainty-aware adaptive acquisition functions by introducing uncertainty penalty terms and multi-fidelity information gain terms based on traditional expected improvement criteria, constructing adaptive sampling strategies that comprehensively consider function improvement potential, prediction reliability, and information acquisition value.
(4) Constructed complete experimental validation systems by establishing real temperature and humidity calibration experimental platforms with spatial layout design of 24 high-precision sensors, obtaining high-quality benchmark data for method validation.

Although this research has achieved significant progress, several limitations still exist that require further improvement in future work. First, current multi-fidelity modeling frameworks are mainly based on linear autoregressive assumptions, which may lead to decreased information fusion accuracy between different fidelity levels for complex temperature and humidity fields with strong nonlinear coupling relationships, affecting overall optimization effectiveness. Second, existing uncertainty quantification methods assume that various uncertainty sources are mutually independent, but in actual engineering environments, model uncertainty, parameter uncertainty, and observation uncertainty often exhibit complex correlations, whose influence mechanisms on overall uncertainty propagation have not been sufficiently studied and quantified. Finally, the proposed method still has optimization space in computational complexity aspects, especially when processing large-scale calibration point candidate spaces, where training and inference processes of multi-fidelity Gaussian process models require substantial computational resources, limiting the method’s applicability to real-time calibration systems.

## Figures and Tables

**Table 1:** Comparison of computational accuracy and efficiency under different grid densities.

Element Sizes (mm)	Number of Elements	Temperature (°C)	%RH
25.0	237,573	40.82	1.13
20.0	413,177	40.56	1.12
17.0	559,989	40.38	1.18
15.0	720,479	40.21	0.98
12.0	884,854	40.09	0.99
10.0	1,127,909	40.10	0.98

**Table 2:** Comparison of optimal calibration point combinations and performance of various methods.

Method	Temperature Calibration Point	Humidity Calibration Point	Temperature Uniformity	Humidity Uniformity	Confidence Interval Coverage Rate
Exhaustive experimental method	[8, 25, 42]	[15, 50, 85]	0.156	2.47	-
GP-MFBO	[9, 26, 41]	[16, 51, 83]	0.149	2.38	94.2
Standard Gaussian process	[11, 24, 44]	[18, 48, 82]	0.127	1.95	86.8
Co-Kriging	[10, 28, 40]	[20, 52, 80]	0.135	2.12	89.3
Two-stage optimization	[12, 27, 43]	[17, 49, 84]	0.119	1.87	82.5
Polynomial regression	[13, 30, 39]	[22, 47, 78]	0.108	1.64	75.1
Single-fidelity calibration	[10, 30, 45]	[20, 50, 80]	0.082	1.35	-

Note: Temperature and humidity uniformity scores are computed using Equations (5) and (6), respectively. Higher scores indicate better spatial uniformity (smaller maximum deviations from setpoints). Confidence interval coverage rate reflects the reliability of uncertainty quantification—higher values indicate more trustworthy predictions.

## Data Availability

The dataset is available on request from the authors.
